# Interstitial Ectopic Pregnancy—Case Reports and Medical Management

**DOI:** 10.3390/medicina59020233

**Published:** 2023-01-26

**Authors:** Małgorzata Kampioni, Karolina Chmaj-Wierzchowska, Katarzyna Wszołek, Maciej Wilczak

**Affiliations:** Department of Maternal and Child Health, Poznan University of Medical Sciences, Polna 33, 69-535 Poznan, Poland

**Keywords:** pregnancy, ectopic, laparoscopy, clinical decision making

## Abstract

The term intramural (interstitial) ectopic pregnancy refers to a pregnancy developing outside the uterine cavity, with a gestational sac implanted into the interstitial part of the Fallopian tube, surrounded by a layer of the myometrium. The prevalence rate of interstitial pregnancy (IP) is 2–4% of all ectopic pregnancies. Surgery is the primary treatment for interstitial ectopic pregnancy; the pharmacological management of ectopic pregnancy, including IP, in asymptomatic patients includes systemic administration of methotrexate. In this report, we present two cases of this rare pregnancy type, reviewing our management technique and treatment ways presented in the literature. In our patients, the management was initially conservative and included methotrexate, administered as intravenous bolus injection, regular beta-human chorionic gonadotropins (β-HCG) level measurements in peripheral blood, and monitoring of the patient’s general condition. Due to signs of intra-abdominal bleeding in patient A and inadequate β-HCG level reduction in patient B, both patients eventually underwent laparoscopic cornual resection. Pregnancy, implanted into the interstitial part of the Fallopian tube and surrounded by myometrial tissue with myometrial invasion of the trophoblast, poses a serious diagnostic challenge to modern gynecology due to particularly low sensitivity and specificity of symptoms, and may require both pharmacological and surgical treatment.

## 1. Introduction

The term intramural (interstitial) ectopic pregnancy refers to pregnancy developing outside the uterine cavity, with a gestational sac implanted into the interstitial part of the Fallopian tube, surrounded by a layer of the myometrium, i.e., the middle uterine wall layer, composed mainly of smooth muscle cells (myocytes), as well as the supporting interstitial and vascular tissue [1,2,3]. The interstitial part of the Fallopian tube is approximately 1–2 cm long and 0.7 mm wide [1]. According to the literature, cornual pregnancy specifically refers to the presence of a gestational sac within a rudimentary uterine horn, a unicornuate uterus, the cornua of a bicornuate uterus, or a septate uterus [4,5].

The prevalence rate of interstitial pregnancy (IP) is estimated to be 2–4% of all ectopic pregnancies by most authors [2,3,5,6,7,8,9] and may range from 6–8%, according to some studies [10]. Due to particularly low sensitivity and specificity of symptoms, it poses one of the largest diagnostic and treatment challenges in modern gynecology. The symptoms, including pelvic, abdominal, or chest pain, vaginal bleeding, intra-abdominal bleeding, hypovolemic shock or uterine rupture are only manifested after 12 gestational weeks in over 20% of cases. They can be life-threatening, and the condition has a mortality rate of up to 2% [1]. The classic clinical triad of ectopic pregnancy, including abdominal pain, vaginal bleeding, and amenorrhea, is only present in 40% of cornual pregnancies [1,3,4,8,11]. Implantation into the tubal wall and myometrial invasion of the trophoblast significantly impede ultrasound-based differential diagnosis of intrauterine or cornual pregnancy [3,8,12,13,14].

The ultrasound diagnostic criteria, developed to identify intramural pregnancy, include [3,8,12,13,14,15]:

- An empty uterine cavity;
- A chorionic sac located eccentrically and at >1 cm from the lateral edge of the uterine cavity;
- A thin (<5 mm) myometrial layer surrounding the chorionic sac;
- The interstitial line sign;
- No double decidual sac sign, typically seen in the intrauterine pregnancy.

The treatment strategies should be individualized, but surgery is still the main treatment of interstitial ectopic pregnancy [9,14]. A number of laparoscopic or laparotomic techniques are available, including cornual resection, salpingectomy, cornuostomy, or hysterectomy. Due to significant advances in endoscopic surgery in recent years, laparoscopic techniques are currently the treatment of choice in the IP [9,10,16] and is preferable to an open approach [15] with laparoscopic cornuotomy or cornual wedge resection [17]. The choice depends on the patient’s condition, availability of medical equipment, and surgical skills of a gynecologist [9,18,19], but laparoscopy has replaced surgical treatments used previously, which included uterine horn resection or even hysterectomy [20].

The pharmacological management of an ectopic pregnancy, including IP, in asymptomatic patients includes the systemic administration of methotrexate (MTX). However, in cases of an IP > 5 cm in diameter, this method fails in 9–65% of cases [19]. In the general population, the failure percentage is estimated to be 25% and additional, surgical treatment is often needed [18]. MTX can also be administered directly into the gestational sac during a local hysteroscopic injection in patients diagnosed at an early stage of IP [21]. This method is described as effective and allows patients to avoid a surgical scar on the uterine muscle [18].

The aim of this study is to present two cases of uterine horn pregnancy to discuss the complexity of the issue and to share our experience in this field as well as review the literature to gain an indication of the different treatment methods.

## 2. Presentation of Case Reports

### 2.1. Case 1 (Patient A)

**Figure 1:**
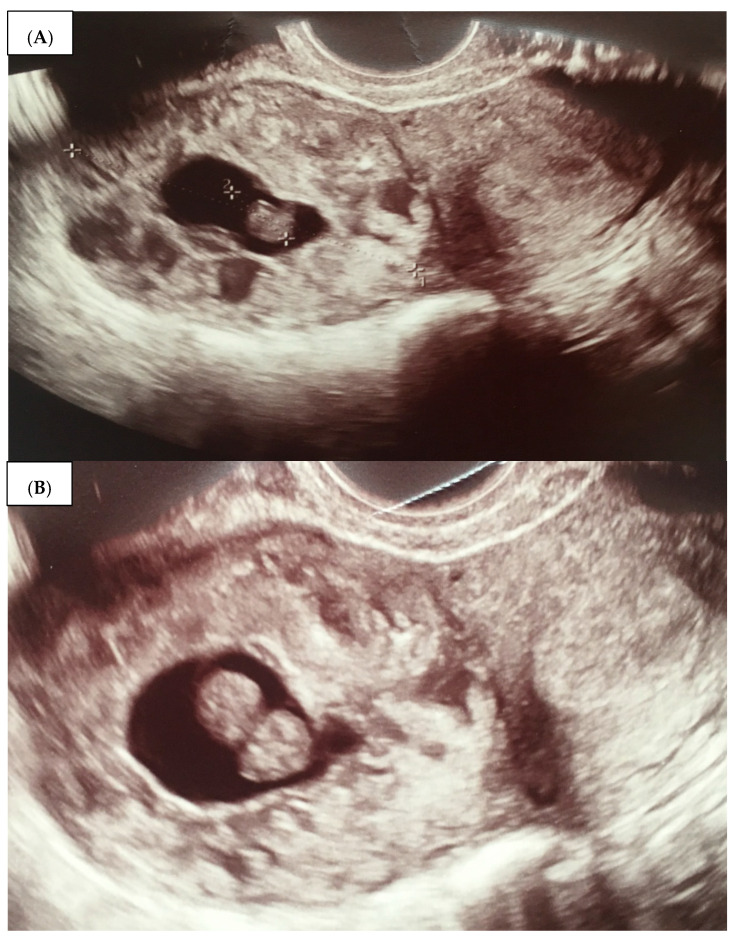
(**A**,**B**)—Patient A—a 2D transvaginal ultrasound of an interstitial ectopic pregnancy within the right uterine horn in different dimensions.

Patient A, a 17-year-old primiparous woman (G0P0A0), was admitted to the Department of Maternal and Child Health, Obstetrics and Gynecology University Hospital, Poznan University of Medical Sciences on 31 July 2019. She was referred by her gynecologist, with an ambulatory diagnosis of 10 weeks of ectopic IP located within the right uterine horn. This diagnosis was confirmed during hospitalization. The β-HCG level on admission was 22,344 mIU/mL. On admission, the patient reported blood-stained vaginal discharge and the absence of other symptoms. The vaginal examination carried out following her giving consent and in the presence of her legal guardian yielded the following findings: ectocervix small, clear and smooth with a punctuate os; moderate amount of dark bloody discharge; uterine body anteroflexed, round, normal in size and mobility; ovaries and Fallopian tubes normal on palpation; no pelvic masses; negative peritoneal signs. A transvaginal ultrasound revealed a uterine body sized 56 × 32 mm, homogeneity in echotexture, and endometrium thickness up to 10 mm. A gestational sac (GS) (15.5 mm in diameter—4w6d, with an embryo crown rump length—CRL = 12 mm − 7w3d) was located interstitially in the right uterine corn, near to the right proximal tubal ostium. Fetal heart rate was 120 bpm. The ovaries appeared normal. There was no free fluid within the cul-de-sac (Figure 1A,B).

Following clinical assessment and based on the wishes expressed by the patient and her legal guardian, pharmacological treatment with methotrexate and leucovorin was started, in line with current guidelines. Methotrexate (100 mg) was administered on 2 August 2019, 5 August 2019, 8 August 2019, and 11 August 2019, followed by oral leucovorin 15 mg. The β-HCG levels were determined accordingly, and the results are shown in Table 1.

**Figure 2:**
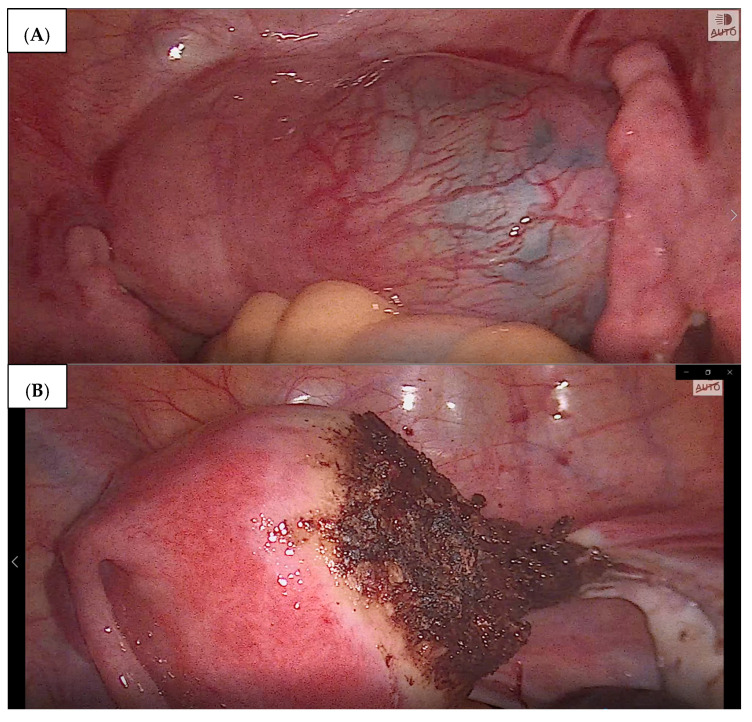
(**A**) Patient A—interstitial ectopic pregnancy within the right uterine horn, viewed from the abdominal cavity. (**B**)—Patient A—Postoperative view following the cornual resection due to interstitial pregnancy. Hemostatic effect after the minimally invasive surgery.

The follow-up ultrasound on 8 August 2019 confirmed the embryo demise within the right uterine horn. Despite receiving a satisfactory response to the systemic treatment with methotrexate, a surgical intervention followed, due to signs of intra-abdominal bleeding found on ultrasound and increasing abdominal pain. On 16 August 2019, the patient underwent a laparoscopy. Intraoperative findings included an enlarged right uterine horn which was approx. 3 cm in diameter. It contained the ectopic gestational sac and, as a result, was significantly hyperemic and swollen. Right salpingectomy was performed, followed by bipolar cautery along the margin of the ectopic gestational sac, and right cornual resection was performed (with morcellation). The stages of the surgery are shown in Figure 2A,B.

The serum β-HCG level on 19 August 2019 was 232 mIU/mL. The postoperative course was uneventful. The patient was discharged home in a stable condition and with recommendations of combined hormonal contraception.

### 2.2. Case 2 (Patient B)

**Figure 3:**
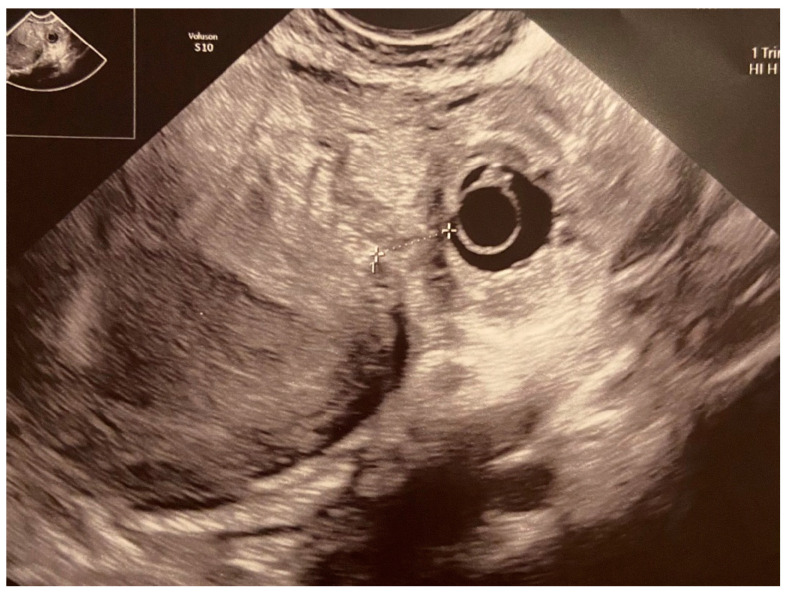
Patient B—2D transvaginal ultrasound of IP within the left uterine horn, cornual bulge.

Patient B, a 33-year-old woman (G1P0A1) presenting with a 7-week interstitial, ectopic pregnancy located in the left uterine horn was admitted to the Department of Maternal and Child Health, Obstetrics and Gynecology University Hospital, Poznan University of Medical Sciences on 8 December 2020. She had undergone laparotomic right salpingo-oophorectomy in 2006 due to mature teratoma of the right ovary. Her last menstruation was on 20 October 2020. The β-HCG level on admission was 5632 mIU/mL. The patient was asymptomatic. The vaginal examination confirmed a small ectocervix, clear and smooth with a punctuate os, normal vaginal discharge, anteroflexed uterine body, normal in size and mobility, left ovary and Fallopian tube normal on palpation, no pelvic masses, and negative peritoneal signs. A transvaginal ultrasound revealed a live intramural pregnancy within the interstitial part of the left Fallopian tube in the left uterine horn, interstitial line sign, myometrial layer surrounding the gestational sac in all projections, cornual bulge, and moderately severe edema where the trophoblast invaded the myometrium. There was also quite significant vascular proliferation within the enlarged uterine horn. A pseudogestational sac, 3 mm in diameter, was found within the uterine cavity. The mass of a total size of 11 mm presented with a detectable fetal heart rate and CRL of 2 mm. Its interstitial location was confirmed. The 6 mm wide chorionic ring containing the yolk sac was imaged. The ovaries appeared normal. There was no free fluid within the cul-de-sac (Figure 3).

Following clinical assessment and a case manifestation, based on the patient’s wishes, pharmacological treatment with MTX and leucovorin was started, in line with current guidelines. MTX (100 mg) was administered on 8 December 2020 and 11 December 2020, followed by oral leucovorin 15 mg. The β-HCG levels were determined accordingly, and the results are shown in Table 2.

The follow-up ultrasound on 14 December 2019 confirmed the embryo demise within the left uterine horn. Due to an unsatisfactory response to pharmacological treatment, a surgery was offered, and the patient consented. On 15 December 2020, laparoscopy was carried out. The procedure revealed a left uterine horn with a tubal fragment preserved after the previous surgery, with tumor-like appearance, 4 cm in diameter, with a soft and heavily vascularized structure. The gestational sac with the fragment of the uterine horn and tubal stump were dissected from the uterine body and resected. Hemostasis was achieved by cautery. The postoperative course was uneventful.

The patient was discharged home on 17 December 2020 in a stable condition with the recommendation of using combined hormonal contraception.

## 3. Discussion

IP may cause life-threatening complications, as a rare and highly dangerous form of ectopic pregnancy, with its lack of specific manifestations, and so its early diagnosis is crucial. Traditionally, the treatment of IP has been surgical and may include hysterectomy or cornual resection by laparotomy or laparoscopy [1,4,9,12,18,19,20].

The advances in minimally invasive surgery have provided more therapeutic options for the treatment of ectopic pregnancies, including the combination of systemic and local hysteroscopic administration of MTX [9,14,20,21,22,23,24]. This is an interesting and promising approach, but this type of treatment requires both implementation in the clinics (including the equipment) and skilled gynecologists to be carried out safely.

Surgical laparotomy is the only appropriate route in the case of unstable hemodynamic women with a suspicion of rupture or recurrent IP [24]. More conservative surgical approaches have been proposed, and currently laparoscopy is the most commonly adopted technique of elective surgery [12]. In the case of Patient A, the decision for surgical management, mentioned as one of the treatment options, was made based on intra-abdominal bleeding exponents shown on ultrasound imaging and peritoneal symptoms. Despite the implementation of systemic MTX treatment, with βhCG showing a decreasing trend, there were symptoms of IP rupture and incipient hypovolemic shock.

Cornual or minicornual resection can be performed in the case of a viable IP with a history of failed therapeutic strategy [25] instead of a cornuostomy that could be adopted with an IP of less than 4 cm in diameter [26]. In the last few years, more conservative surgical alternatives, such as cornuostomy rather than cornuectomy, have been introduced to better preserve uterine integrity for future fertility [25,26].

Some cases of laparoscopic cornuostomy have been reported in the literature [2,3,6,27]. However, patients with a history of ipsilateral salpingectomy should be cautioned regarding the possibility of IP. Laparoscopic cornuostomy appears to be an appropriate treatment for IP in patients wishing to preserve fertility, and the use of concomitant prophylactic MTX may reduce the risk of persistent ectopic pregnancy, especially among patients with ruptured masses and high β-HCG levels [28]. Po et al. [17] stated that clinicians may perform either laparoscopic cornuotomy or cornual wedge resection because both procedures have comparable results, but this summary statement was rated as conditional and low in the GRADE evidence quality.

The treatment should be personalized in a way that considers the obstetric history of the patients, the gestational age at the diagnosis, and their desire for future pregnancies [9,14]. Stabile et al. [14] proposed a multidose MTX intramuscular regimen, combined with mifepristone (600 mg orally), in asymptomatic women with low serum levels of β-HCG at an early gestational age. It can be also considered in asymptomatic women with a strong motivation for future conceptions, although in the case of high serum levels of β-HCG, additional dose(s) of MTX may be necessary. The overall efficacy of a single MTX dose is estimated to be 65–95%, and such a variability is due to several factors: the baseline level of β-HCG (the lower the level, the higher the efficacy of the treatment), the rate of serum β-HCG growth over 48 h prior to MTX administration, the visibility of specific elements of the fetal egg on ultrasound, and the rate of decrease in β-HCG levels after the implementation of the pharmacological treatment [29]. Our patient described as a B, despite of the implementation of MTX treatment and leucovorin, presented a non-satisfactory response to drug treatment (β-HCG serum level decreased <15% of the initial level). After discussing possible management routes, the patient consented for a laparoscopic surgery.

Tulandi and Al-Jaroudi [30] discussed the management of 32 interstitial pregnancy cases. Eight women were treated with MTX either systemically (n = 4), locally under ultrasonographic guidance (n = 2), or under laparoscopic guidance (n = 2). Eleven patients were treated by laparoscopy and 13 by laparotomy. Systemic MTX treatment failed in three patients, and they required surgery. Persistently elevated serum β-HCG levels were found in one patient after laparoscopic cornual excision, and she was successfully treated with MTX. Subsequent pregnancy was achieved in 10 patients. No uterine rupture was encountered during pregnancy or labor [30].

Alagbe et al. [31] reported a case of a right interstitial ectopic pregnancy diagnosed in a 39-year-old woman. The gestational sac diameter was 2.7 cm, equivalent to 7 weeks of gestation. The patient was admitted for medical management (using intramuscular MTX 75 mg) and serial ultrasound monitoring. The ultrasound revealed a persistent gestational sac on the 8th day, following MTX injection. On day 10, however, the gestational sac completely disappeared [31].

Dagar et al. [1] retrospectively analyzed three cases of interstitial pregnancy. In the third case they combined both modalities, local and systemic MTX administration, along with local KCl injection. This was one of the few case reports of such an approach.

The combined method, described as hysteroscopy-assisted laparoscopy, was described by some authors as an alternative minimally invasive approach that could be appropriate in some patients with IP. The prerequisites included early recognition of the abnormality and the woman’s hemodynamically stable condition [32,33]. Katz et al. [32] presented two cases of patients with diagnosed IP who were successfully treated with laparoscopic-assisted hysteroscopy. The evacuation of the gestational sac was carried out transvaginally under laparoscopic supervision. Similarly, Feng et al. [33] described a case of a patient diagnosed with IP. They were initially unsuccessfully treated with MTX, and then subsequently with laparoscopic-assisted hysteroscopy [33]. In all the cases presented, cornual resection was not necessary, which is undoubtedly an advantage of this method [32,33].

Kahramanoglu et al. [34] presented four cases of patients with IP. In that series, each patient needed a different treatment modality—a single dose of MTX, laparotomy, hysteroscopy followed by vacuum aspiration, and vacuum aspiration under laparoscopic control. The treatments depended on the patients’ presenting symptoms, β-HCG levels, and ultrasound images. This article perfectly illustrates the complexity of the IP [34].

In 2021, Marchand et al., presented a comprehensive systematic review [35] and a meta-analysis [36] of the patients diagnosed with IP in which they compared the outcomes of the laparoscopic surgery versus laparotomy treatment. The first paper included one case series study, one cross-sectional study, and four retrospective cohort studies with 70 cases of IP in the laparoscopic surgery group and 83 cases in the laparotomy surgery group [35]. The authors concluded that laparoscopic management was associated with a shorter postoperative hospital stay.

In the mentioned meta-analysis [36] and the review [35], the authors compared the effects of laparoscopic versus laparotomy treatment in 855 women with IP. They included 65 case reports, 23 cohort studies, 6 case series, and 2 case–control studies, meeting the search criteria. They found that 723 women underwent laparoscopy, while 132 were treated with laparotomy [35]. The analysis demonstrated more favorable outcomes of laparoscopy vs. laparotomy, i.e., less bleeding during surgery, shorter duration of the procedure and the hospital stay, and a higher risk of rupture of ectopic pregnancy when laparotomy was performed. In conclusion, the authors suggested laparoscopy as the first-choice method when a surgical approach is necessary in patients diagnosed with IP [35].

The condition of ectopic pregnancy can develop rapidly, leading to hemodynamic instability and death. Thus, it is important to promptly recognize the classic ultrasound presentation. The awareness of appropriate diagnostic approaches, differential diagnoses as well as conservative and surgical treatment methods are equally vital [1,9,14,15,16,17,18,29,32,33,34,35,36].

It is difficult to identify a single management method of uterine horn pregnancy due to the highly variable response to treatment and the dynamics of the development of symptoms, which can threaten the health and life of patients. In the literature, particular management approaches have been proposed, but the level of evidence for them was low [15,17].

## 4. Conclusions

The effectiveness of pharmacological treatment depends on a variety of factors, and the patient should remain under careful observation until a treatment course is completed. The dynamic development of endoscopic surgery in recent years has made the laparoscopic techniques the treatment of choice in IP. The development of minimally invasive techniques allows for less burdensome treatment of patients with IP, but requires experience in the use of this technique in order to treat the ectopic pregnancy.

## Figures and Tables

**Table 1:** Serum β-HCG levels in the patient A, July/August 2019.

Date	31 July	5 August	8 August	9 August	14 August	16 August
β-HCG (mIU/mL)	22,344	29,502	15,735	17,138	13,171	6463

**Table 2:** Serum β-HCG levels in the patient B, December 2020.

Date	8 December	11 December	14 December
β-HCG (mIU/mL)	5632	5927	5739

## Data Availability

Not applicable.
